# A new method for oral cancer biomarkers detection with a non-invasive cyto-salivary sampling and rapid-highly sensitive ELISA immunoassay: a pilot study in humans

**DOI:** 10.3389/fimmu.2023.1216107

**Published:** 2023-07-06

**Authors:** Federico Rebaudi, Alfredo De Rosa, Marco Greppi, Roberto Pistilli, Resi Pucci, Flavio Andrea Govoni, Paolo Iacoviello, Francesco Broccolo, Giuseppe Tomasello, Silvia Pesce, Francesco Laganà, Bernardo Bianchi, Francesca Di Gaudio, Alberto Rebaudi, Emanuela Marcenaro

**Affiliations:** ^1^Department of Experimental Medicine (DIMES), University of Genova, Genova, Italy; ^2^Multidisciplinary Department of Medical-Surgical and Dental Specialties, University of Campania “Luigi Vanvitelli”, Napoli, Italy; ^3^Oral and Maxillofacial Surgery, San Camillo-Forlanini Hospital, Roma, Italy; ^4^Department of Maxillofacial and Plastic Reconstructive Surgery, E.O. Ospedali Galliera, Genova, Italy; ^5^Department of Biological and Environmental Sciences and Technologies, University of Salento, Lecce, Italy; ^6^Private Practice, Lugano, Switzerland; ^7^IRCCS Ospedale San Martino, Unità Operativa Complessa di Chirurgia Maxillofacciale e Odontoiatra, Genova, Italy; ^8^Department of Health Promotion, Mother and Child Care, Internal Medicine and Medical Specialties, University of Palermo, CQRC (Quality Control and Chemical Risk) Hospital Company, Hospitals Reunited Villa Sofia Cervello, Palermo, Italy; ^9^Private Practice, President of Bio.C.R.A. (Biomaterials Clinical-Histological Research Association), Genova, Italy; ^10^IRCCS Ospedale Policlinico San Martino, Genova, Italy

**Keywords:** oral cancer, screening, tumor biomarkers, natural killer cells, immunotherapy, ELISA immunoassay, cytobrush, immune checkpoints

## Abstract

**Introduction:**

Oral squamous cell carcinoma (OSCC) accounts for approximately 90% of oral malignancies and has a 5-year mortality rate close to 50%. A consistent part (70%) of all oral cancers is diagnosed at an advanced stage since available screening techniques are ineffective. Therefore, it would be urgent to improve them. The diagnostic gold standard is tissue biopsy with histological and immunohistochemical assessment. This method presents some limitations. Biopsy is invasive and the histopathological evaluation is semi-quantitative, and the absolute abundance of the target cannot be reliably determined. In addition, tissue is highly processed and may lead to loss of information of the natural state. The search for classical and new clinical biomarkers on fragments of tissue/cells collected with a cytobrush is a highly hopeful technique for early detection and diagnosis of OSCC, because of its non-invasive sampling and easy collection method.

**Methods:**

Here we analyzed cytobrush biopsies samples collected from the oral cavity of 15 patients with already diagnosed OSCC by applying an innovative high-sensitivity ELISA technique, in order to verify if this approach may provide useful information for detection, diagnosis, and prognosis of OSCC. To this end, we selected six biomarkers, already used in clinical practice for the diagnosis of OSCC (EGFR, Ki67, p53) or selected based on recent scientific and clinical data which indicate their presence or over-expression in cells undergoing transformation and their role as possible molecular targets in immunecheckpoints blockade therapies (PD-L1, HLA-E, B7-H6).

**Results:**

The selected tumor biomarkers were highly expressed in the tumor core, while were virtually negative in healthy tissue collected from the same patients. These differences were highly statistically significant and consistent with those obtained using the gold standard test clearly indicating that the proposed approach, i.e. analysis of biomarkers by a custom ELISA technique, is strongly reliable.

**Discussion:**

These preliminary data suggest that this non-invasive rapid phenotyping technique could be useful as a screening tool for phenotyping oral lesions and support clinical practice by precise indications on the characteristics of the lesion, also with a view to the application of new anti-tumor treatments, such as immunotherapy, aimed at OSCC patients.

## 1 Introduction

Oral cancer is the sixth most common malignancy worldwide (1). Globally, over 400,000 new cases of oral cancer are diagnosed each year. The incidence increases with age, even though cases in subjects younger than 40 years are growing. In more than 90% of cases, oral cancers are squamous cell carcinomas (OSCCs) with a tendency to lymphatic and metastatic spreading (2–5). Oral cancer has poor prognosis, with a 40% overall 5-year survival rate but when diagnosed in the early stage, survival rates can exceed 80% (6–8). The high mortality rate associated with this tumor is attributable to many factors, including generic and non-specific symptoms and absence of validated screening strategies that allow for early diagnosis, thusmost of the oral cancers (70%) are diagnosed late at an advanced stage (II-III-IV) (9–14).

Treatments for advanced stage oral cancer require mutilating interventions and the evolution of the disease leads to a very low quality of life. Oral cancer should be detected at a very early stage which will improve the effectiveness of therapies, to reduce mortality and morbidity (13, 15–18).

Up to now, there are no scientifically approved systems able to detect a lesion in the early phases of tumor transformation (19) other than the conventional clinical visual oral examination (VOE), with the aid of magnifying optics and fluorescent systems as well as the palpation of the oral cavity and neck to detect abnormalities. Currently, surgical biopsy is the most effective method to collect tissue useful for diagnosis (20) and it represents the gold standard as a diagnostic method. However, this approach is invasive and does not allow an early diagnosis because it is done on lesions that are visible at clinical examination. Moreover, the histopathological specimen examination is a method with some limitations because it is semi-quantitative, and the absolute abundance of the target cannot be reliably determined. Furthermore, the tissue is highly processed and may lead to loss of information of the natural state (21). Currently, one the main aims of clinical research is the identification of biomarkers to monitor and discover effective therapeutic strategies. In other fields of medicine, such as gynecology, the early diagnosis of cervical cancer through cytobrush biopsy, PAP test and HPV test, has proven to be very effective in reducing mortality, morbidity, and costs for the community (22). Oral cytobrush was proposed to collect tissue particles, cells, and a small amount of saliva at the same time (23). For this reason, it is a promising method in identifying early disease onset. Recently, the ability to detect molecules with cytobrush from patients with head and neck cancer (24) provided along with saliva samples (25) was claimed to be a unique opportunity to develop noninvasive diagnosis. Nevertheless, no single biomolecule has been shown to meet the real-world requirement for high accuracy in identifying early disease onset, suggesting that multiple biomarkers are needed for high accuracy and sensitivity in detecting OSCC (26). Surgical biopsy is an invasive diagnostic method, while cytobrush technique is suitable for the screening of pathological conditions considering its minimal invasiveness (23). Therefore, it is important to evaluate whether the cytobrush might be sufficient to be used as a reliable standard method to aid in the diagnosis of suspicious oral lesions. To date, the diagnostic precision and accuracy of the cytobrush technique for finding oral cancer biomarkers versus histopathologic diagnosis has not been examined in detail (27).

Our preliminary study aims to establish whether cytobrush biopsy is effective in collecting OSCC selected biomarkers (22, 28–34) from an oral cancer lesion and to evaluate, from a statistical point of view, its accuracy (as diagnostic effectiveness) and precision (as discriminatory effectiveness between the tumor lesion and the surrounding healthy tissue) in order to perform a correct diagnosis by using the Stark Oral Screening^®^ IVD test (Stark S.a.r.l., Principaute de Monaco) and the Femtohunter^®^ device (Stark S.a.r.l.) and comparing its outcome with the biopsy results, taken as the gold standard.

## 2 Materials and methods

To perform our study, we used a diagnostic system consisting of the Femtohunter^®^ device and Stark Oral Screening^®^ IVD test kits.

### 2.1 Subjects

Fifteen patients (9 males, 6 Females) with a newly diagnosed primary OSCC (staged I-IV according to the tumor-node-metastasis-TNM criteria) without prior chemotherapy or radiotherapy were enrolled for the study between May 2022 and April 2023.

### 2.2 Criteria of inclusion

Adult patients (>18years old) diagnosed with oral cancer (confirmed by histological diagnosis), all cancer stages, no previous treatment.

### 2.3 Criteria of exclusion

Patients without a confirmed diagnosis of oral cancer by histology or immunohistochemistry. Brush samples with evident blood traces or pyrogens with quantitative negative channel noise background values equal to or greater than 20. Brush samples with an insufficient cell load expressed by beta-actin channel value less than or equal to Femtohunter F.M. 2.

### 2.4 Samples collection

We collected cytobrush biopsies from the patients and analyzed them for the expression of selected biomarkers with a high-sensitivity fully automated ELISA technique. Patients were asked to rinse their mouths with physiologic solution before performing the cytobrush biopsy retrieval. Two non-invasive cytobrush biopsies were taken from the mouth of each patient with cancer. For each patient, the first sample was obtained from the center of the lesion and the second one from surrounding healthy tissue. Each cytobrush was rubbed applying a mild pressure and a rotation over the area under analysis to collect cells and fragments of tissue exfoliation, while limiting bleeding as much as possible. Cytobrush tips were inserted in sealed Eppendorf vials, cataloged, and stored at 0-4°C and then sent to the lab for analysis in refrigerated boxes.

### 2.5 Selection of biomarkers

We selected six biomarkers:

- EGFR (35–38), p53 (39–41), Ki67 (29, 42–48), already available and consolidated in their diagnostic effectiveness of OSCC;
- PD-L1, B7-H6, HLA-E, selected based on recent scientific and clinical data which indicate their *de novo* or over-expression in cells undergoing transformation as a mechanism of tumor-mediated immune resistance and their role as possible molecular targets in immune checkpoints blockade therapies (30–34).

### 2.6 Biomarkers detection technique

Two different disposable Stark Oral Screening^®^ test kits (Stark S.a.r.l.) were used for the analysis of the biological samples:

- Stark oral screening quantitative metabolic (REF: SOSFMTCKIT) detection of EGFR/p53/Ki67
- Stark oral screening quantitative NK time (REF.SOSBHPDQNT) detection of B7-H6/PD-L1/HLA-E.

The Stark Oral Screening^®^ test is a patient side *in vitro* diagnostics (IVD) and quantitative test based on a bioluminescent signal response. The Stark Oral Screening^®^ test has a very high sensitivity with the following limit of detection value (LOD) “*In vitro*”: LOD = 20 Femtograms/microliter.

Disposable kits are made up of 3 elements:

1) Cytobrush for non-invasive sampling of the biological sample.
2) Reagent slots - A thermoformed tray in two superimposed layers defined in slot caps and test tube slots so designed to keep the solid phase separate from the liquid phase, in order to be able to make the storage at room temperature. The reagent slot has 10 cavities, called “stations” necessary for the correct automatic execution of the ELISA procedure.
3) Slot detector membranes in PVDF - The thermoformed tray has 3 cavities which allow the anchoring of 3 membranes armed with polyclonal antibodies towards markers of interest as well as 1 control membrane.

Disposable kits had to be processed through Femtohunter (Stark Sarl, Principaute de Monaco).

### 2.7 Data preparation

The biological samples were inserted into the respective cavities of the Stark kit reagent slot to identify:

- EGFR, p53, Ki67 markers (Cod kit SOSFMTCKIT)
- B7-H6, PD-L1, HLA-E markers (Cod Kit SOSBHPDQNT)

The reagent slots, loaded with the biological sample from the patient, were inserted into the automatic ELISA developer device (Femtohunter) with chemiluminescent signal and qualitative and quantitative analysis response. The slot membranes, armed with polyclonal antibodies against the marker of interest, were inserted into the automatic developer device.

The automatic procedure took place in 13 development steps: 12 steps included the correct ELISA procedure and 1 step was focused on chemiluminescent signal detection and analysis.

**Figure 1:**
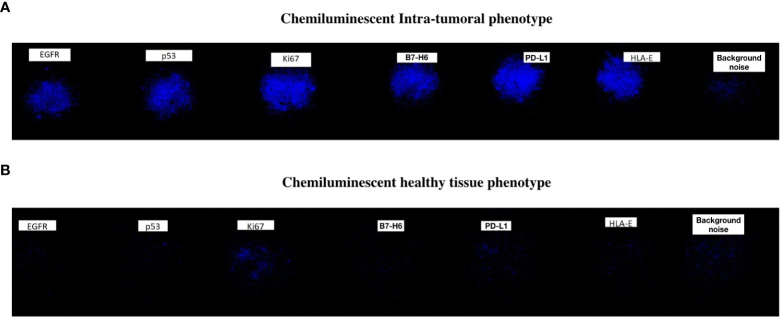
An example of Chemiluminescent Phenotype showing the six selected biomarkers in an intra-tumoral sample **(A)** and healthy tissue sample **(B)**.

Each chemiluminescent signal (S) detected with the Femtohunter device (**Figure 1**) is calibrated at each test by measuring the intensity of the background noise (N), generated by non-specific luminescence on a control PVDF membrane. The N value is then subtracted from the S value present on the PVDF membrane dedicated to the marker. If the result is a positive value (S-N>0) then it assesses that there is a specific signal S for the sought marker, therefore its presence in the sample. The positive value S is then divided by N generating a multiplication factor and allowing the operator to understand how many times the specific signal S is intense compared to the background noise N (Signal/Noise ratio). The obtained S/N value is defined as FM (multiplication factor for the Femtohunter^®^) and included in the Femtohunter^®^ FM patient report. For instance, the value 2.4 EGFR expresses that there is a chemiluminescent signal S on the PVDF dedicated to EGFR greater 2.4 times the background noise N. The test was considered POSITIVE for a malignant tumor lesion when it showed the Femtohunter^®^ FM > 1.2 for all six markers of interest. The test was considered NEGATIVE for a malignant tumor lesion when it showed a Femtohunter^®^ FM < 1.19 for any of the markers of interest.

### 2.8 Statistical methods

First, a power analysis (**Table 1**) was carried out to estimate the minimum number of statistical units necessary to guarantee the test a power of at least 0.8. The significance level chosen is 0,05.

**Table 1:** Power analysis.

	N	Actual Powerb	Test Assumptions			
			Power	Std. Dev.	Effect Size	Sig.
**Test for Mean^a^**	**7**	**0,820**	**0,8**	**14,39**	**1,098**	**0,05**
a. One-sided test.						
b. Based on non-central t distribution.						

The primary outcome identified to perform the power analysis is the parameter PD-L1.

The reasons why we chose the PD-L1 marker are related to its characteristics:

- PD-L1 is also expressed in oral cancer precancerous lesions (49), and it is usually overexpressed in tumor lesion, so it is a statistically strong parameter.
- PD-L1 expression can render the cancer invulnerable to immune attack. In fact, the PD-1/PD-L1 axis plays an important role in oral cancer therapy. Thus, PD-L1 is not only a guidance for the use of immune checkpoint inhibitors (50), but also a potential prognostic indicator for oral cancer (51). Therefore, focusing on its presence in the sample is not only useful for statistical purposes since it could have a prognostic value.

Empirical data, observed in a first pilot sample, were used to identify the expected value for the “PD-L1 tumor center” variable, as no literature data are available. Healthy tissue for the variable PD-L1 has an expected value, by definition, equal to 0, with no standard deviation. Therefore, a one-sample power analysis was performed to compare the expected mean value of “PD-L1 tumor center” with the reference value of 0.

A univariate descriptive analysis of the FM parameters was then carried out by calculating the centrality and variability indexes for quantitative variables. Therefore, nonparametric Wilcoxon tests for paired samples were used to verify whether there was a statistically significant difference, in terms of distribution of EGFR, p53, Ki67, B7-H6, PD-L1 and HLA-E parameter values, between the tumor center and the healthy tissue of the same subjects.

The null hypothesis is H0 = no significant differences in the distribution of parameters between the tumor center and the healthy tissue. An alpha significance level of 0.05 was used in all these analyses.

Healthy patients have not been added to the study, as control is the healthy tissue of the patients themselves, thus making the recruitment of additional patients without pathology superfluous.

In fact, the patients were enrolled in the study because they were diagnosed with OSCC by biopsy, considered as a gold standard, i.e. the most accurate diagnostic examination to confirm a certain diagnostic question, to which every other examination (or any other new examination) must relate to have diagnostic validity (52). Moreover, in this type of statistical evaluation, the use of a cohort as a control would inevitably increase overall bias (i.e. false negatives and false positives).

IBM SPSS Statistics software in version 28 was used for statistical analysis of data.

## 3 Results

### 3.1 Power analysis

The PD-L1 parameter, chosen as the primary outcome for the power analysis, shows in the preliminary data an average value of 15,8 with a standard deviation of 14,39 for the tumor center. While the score for PD-L1 parameter in healthy tissue is by definition equal to 0. The final mean PD-L1 score of the 15 enrolled patients, as evident in the table “FM Descriptive statistics for each parameter and Wilcoxon comparison test”, turns out to be different (i.e. 13.1) from the mean value hypothesized in the power analysis (i.e. 15.8) since the latter, being by definition an analysis that is carried out “a priori”, was carried out on the preliminary data, i.e. on the data relating only to the first 7 patients enrolled (as evidenced by the value “7” in the column called “ N” of the Power analysis table), whose mean and standard deviation were respectively 15.8 and 14.39. A one-tail t-test was used for the single-sample mean (H0: μ = 0; H1: μ > 0) (**Table 1**).

The power analysis showed that the minimum number of statistical units necessary to obtain a power of 0,8 with reference to the PD-L1 parameter is 7 units. Therefore, our sample size can be considered definitely adequate in terms of actual power of the tests and should be able to accurately represent the total population.

### 3.2 Statistical analyses

After sample collection, data was analyzed obtaining the following results:

Intra-tumoral markers (**Table 2**): All samples from the tumor center showed 6 over-expressed markers out of 6 markers, except for two subjects who had only 5 markers out of 6.

**Table 2:** Intra-tumoral markers.

		Frequency	Percent	Cumulative Percent
Markers	5 out of 6	2	13,3	13,3
	6 out of 6	13	86,7	100,0
	Total	15	100,0	

Healthy tissue markers (**Table 3**): All samples from the healthy tissue showed 0 to 3 over-expressed markers out of 6 markers.

**Table 3:** Healthy tissue markers.

		Frequency	Percent	Cumulative Percent
Markers	0 out of 6	5	33,3	33,3
	1 out of 6	7	46,7	80,0
	2 out of 6	2	13,3	93,3
	3 out of 6	1	6,7	100,0
	Total	15	100,0	

FM Descriptive statistics for each parameter and Wilcoxon comparison test (**Table 4**)

**Table 4:** FM Descriptive statistics for each parameter and Wilcoxon comparison test.

	Mean	Standard Deviation	Median	Percentile 25	Percentile 75	p-value
EGFR Tumor tissue	7,7	6,7	5,1	4,8	7,9	0,001***
EGFR Healthy tissue	0,8	2,3	0,0	0,0	0,0	
p53 Tumor tissue	8,0	8,2	5,5	4,3	7,0	<0,001***
p53 Healthy tissue	0,0	0,0	0,0	0,0	0,0	
Ki67 Tumor tissue	9,1	6,1	6,7	5,4	10,5	0,008**
Ki67 Healthy tissue	2,8	4,8	0,0	0,0	4,9	
B7-H6 Tumor tissue	8,0	4,8	6,9	4,6	9,4	0,001***
B7-H6 Healthy tissue	1,4	2,4	0,0	0,0	3,4	
PD-L1 Tumor tissue	13,1	12,4	8,4	5,7	15,8	<0,001***
PD-L1 Healthy tissue	0,0	0,0	0,0	0,0	0,0	
HLA-E Tumor tissue	10,3	6,9	6,9	5,8	15,4	<0,001***
HLA-E Healthy tissue	0,2	0,6	0,0	0,0	0,0	

**Figure 2:**
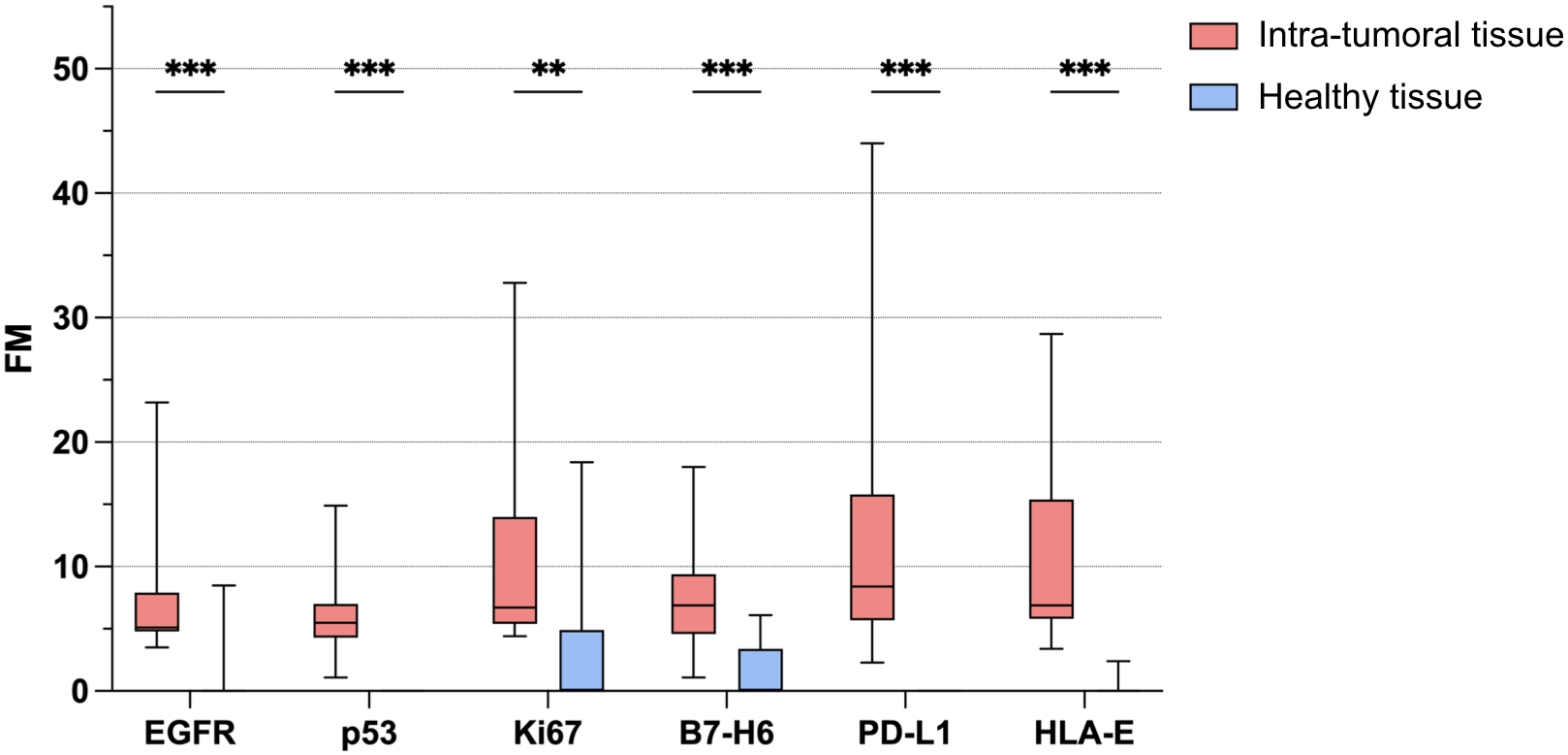
Bio-marker detectability in intra-tumoral and healthy tissue samples. Variation in the FM (moltiplicative factor) of each of the six bio-markers between intra-tumoral (red) and healthy (blue) samples in patients with OSCC (N=15). P value of less than 0.01 (**) and less than 0.001 (***) was considered statistically significant.

The Wilcoxon non parametrical paired test is statistically significant for all six analyzed parameters (EGFR p:0,001; p53 p:<0.001; Ki67 p:0.008; B7-H6 p:0.001; PD-L1 p:<0.001; HLA-E p:<0.001). Therefore, we can reject the null hypothesis that there is no significant difference in the distribution of parameters between the tumor center and healthy tissue. Specifically, all the six parameters have significantly higher values in the tumor center than in healthy tissue (**Figure 2**).

The bioluminescent phenotype observed in the two different sampling sites (healthy tissue and center of the lesion) differs not only in signal quality but also in quantitative intensity with a signal that is on average 213% more intense in the center of the tumor than in the health tissue, confirming a marked over-expression of markers in the tumor center and a sharp decay or a disappearance of the signal in healthy tissues. This result was independent of tumor staging.

These results are consistent with those obtained using the gold standard test and clearly indicate that the proposed approach (analysis of biomarkers by an automated proprietary ELISA technique) is highly reliable, being able to detect tumor markers only in the core of the tumor and not in the healthy tissue.

## 4 Discussion

The Femtohunter^®^ is an automatic ELISA developer device that automatically performs the chemiluminescence analysis on samples taken by cytobrush and submitted to the Stark Oral Screening^®^ IVD test, provides the results as a graphical image within 60 minutes, as well as the analytical data of the markers and prints a patient report. The Stark Oral Screening^®^ test is a patient side *in vitro* diagnostics (IVD) and quantitative test based on a bioluminescent signal response. The Stark Oral Screening^®^ test has a very high sensitivity with the following limit of detection (LOD) values of 20 Femtograms/microliter.The aim of the present study was to verify whether the detection of appropriately selected biomarkers in cytobrush biopsies samples by the Femtohunter can discriminate the lesions of OSCC from the surrounding healthy tissue. To this end, we have selected 6 biomarkers, some of these already used in clinical practice for the diagnosis of OSCC (EGFR, p53, Ki67), other selected based on recent scientific and clinical data which indicate their *de novo-* or over-expression in cells undergoing transformation and their role as possible molecular targets in immune checkpoints blockade therapies (B7-H6, PD-L1, HLA-E). The choice of these markers is based on recent evidence of the important role that the immune system plays in preventing but also in promoting the development of tumors. Indeed, resistance mechanisms are commonly employed by tumors in response to immune pressures exerted by effector cells, such as natural killer (NK) cells and cytotoxic T cells. Among such resistance mechanisms, increased expression of inhibitory receptors on CD8^+^ T cells and Natural killer (NK) cells constrains their antitumor cytotoxic potential (53, 54). Monoclonal Antibodies (mAbs) that abrogate these inhibitory interactions between immune checkpoint receptors and their ligands have transformed the therapeutic landscape for treatment of solid tumors (54–56). NK cells are lymphocytes of the innate immune system that sense target cells through a panel of activating and inhibitory receptors expressed at their surface. Integration of the opposing signals transduced by the engagement of such receptors defines the functional outcome for NK cells (57). Inhibitory receptors include the KIRs and CD94/NKG2A molecules, and their interaction with classical (HLA-I molecules) and non-classical (HLA-E) on target cells prevents activation of NK cells. Thus, downregulation of normally ubiquitously expressed HLA-I molecules on target cells activates NK cells, a process coined as “missing self” recognition, while the maintaining of HLA-I expression on the tumor surface blocks NK cell ability to kill the tumor. Recently, the PD-1 receptor, originally identified on T cells, has been described on a subset NK cells as an additional inhibitory receptor that can block NK cell function against tumor cells expressing the specific ligands PD-L1 and PD-L2. Thus, in pathological conditions, these inhibitory receptors (primarily NKG2A and PD-1) can function as immune checkpoints by blocking the functional activity of NK cells against tumor cells expressing the relative ligands. These NK cell impairments can be rescued using specific mAbs able to disrupt the receptors/ligands interactions, thus demonstrating a role for these inhibitory receptors as true immune checkpoints (clinicaltrials.gov). For these reasons, these receptors, mainly NKG2A and PD-1, and their relative ligands (HLA-E and PD-L1) are considered possible molecular targets in the immune checkpoint blockade immunotherapy. HLA-E is a non-classical MHC class I molecule that is ubiquitously expressed on hematopoietic cells at low abundance on the cell surface and is sensitive to inflammatory signals. HLA-E binds to the heterodimeric complex CD94/NKG2A (58–61). A humanised mAb binding to the NKG2A receptor, Monalizumab, has been developed, and numerous clinical trials are ongoing across multiple tumor indications (clinicaltrials.gov). Monalizumab can be potentially used in the treatment of oral cancers (30, 61). *In vitro* blockade of NKG2A, alone or in combination with targeting the PD-1 pathway, stimulates NK cell functions but is collectively required to stimulate a strong CD8+ T cell response to HLA-E^+^ PD-L1^+^ tumors. The combined administration of anti-NKG2A and anti-PD-L1 blocking antibodies unleashes NK and CD8^+^ T cells and subsequently slows tumor progression in mouse models and preliminary analyses suggest *in vivo* efficacy of Monalizumab when in combination with the EGF receptor (EGFR) blockade antibody (Cetuximab) in recurrent/metastatic squamous cell carcinoma of the head and neck (HNSCC) (30). PD-1/PD-L1 blockade has been tested in clinical trials for various malignancies including metastatic oral carcinoma, with significant response rates and limited side effects. Immunotherapies targeting the PD-1/PD-L1 pathway have particularly proven effective in controlling tumor growth through the reinvigoration of CD8^+^ T cells and/or NK cells across numerous tumor settings, including oral cancer (30). PD-L1 expression has also been proposed as a prognostic marker for different types of cancers with mixed results. Based on these data and considering the high expression of HLA-E and PD-L1 on the surface of oral cancer cells (30), we selected these two molecules as potential OSCC biomarkers. Regarding the selection of B7-H6 as a possible additional marker of oral cancer, we should consider that NK cells (and other immune cells) also express a series of activating receptors, including the so-called Natural Cytotoxicity Receptors (NCR), that include NKp46 (NCR1), NKp44 (NCR2) and NKp30 (NCR3). NKp30 was identified at the end of the 1990s as a novel 30-kDa triggering receptor expressed by all resting and activated human NK cells (57, 62). Cells expressing NKp30 ligands are sensitive to the cytotoxicity of human NK cells. The identification of B7-H6 as a counter structure of the NCR3 NKp30 shed light on the molecular basis of NK cell immunosurveillance. B7-H6, a member of the B7 family of immune modulators, is expressed in a variety of tumor cell types while minimally or not expressed in normal tissues. Expression of B7-H6 on the tumor cell surface can markedly enhance the sensitivity of tumor cells to NK cells. Several studies suggest that NK cells can potentially eliminate B7-H6-positive tumor cells in cancer patients. However, as clinically observed, most of the human tumors are found to be B7-H6+ rather than B7-H6−, which suggests the functional compromise of the B7-H6 ligand-NKp30 receptor system in cancer patients to permit the growth of B7-H6+ tumor cells (33). A study evaluating different doses of BI 76504, an anti-B7-H6/Anti-CD3 bispecific antibody, given alone and given with Ezabenlimab (an anti-PD-1 drug) to patients with advanced solid tumors (including oral cancers) having the B7-H6 marker is currently recruiting participants (clinicaltrials.gov). BI 765049 is an immunoglobulin G (IgG)-like bispecific T-cell engaging antibody directed against both NCR3 (NKp30) ligand 1 (NCR3LG1; B7-H6) and T-cell surface antigen CD3, with potential immunostimulating and antineoplastic activities. Upon administration, anti-B7-H6/anti-CD3 bispecific antibody BI 765049 targets and binds to both B7-H6 on tumor cells and CD3 on T cells. This results in the cross-linking of B7-H6-expressing tumor cells and T-cells, redirects cytotoxic T lymphocytes (CTLs) to B7-H6-expressing tumor cells, which leads to the CTL-mediated cell death of B7-H6-expressing tumor cells. Thus, considering these clinical data, and the presence of B7-H6 in certain cancers including oral cancers, and its ability to modulate immune cell function that can be exploited in immunotherapeutic approaches, we selected this molecule as a further OSCC biomarker to detect in our system. To confirm the validity of this technique, multiple cytobrush samples were collected from the oral cavity of 15 patients with already diagnosed OSCC in order to search the selected markers. Patients had diagnosed oral cancers ranging from stage I to IV, representing the full spectrum of OSCC. Cytobrush samples analyzed with the novel high-sensitivity ELISA technique demonstrated reliability, specificity, discriminatory value, and low cost. These results are consistent with those obtained using the gold standard test and clearly indicate that the proposed approach is highly reliable, being able to detect tumor markers only in the core of the tumor and not in the healthy tissue. This innovative approach could pave the way for the realization of a new method of screening and phenotyping of oral lesions with advantages on patients’ health and possible simplification of screening procedures for obtaining early diagnosis. Many patients are currently being discharged after a negative biopsy result of a suspected precancerous lesion or potentially malignant oral disorders. On the contrary, these patients should be placed on a regular follow-up schedule to prevent a precancerous lesion from turning into cancer over time. Although biopsy with histological examination is universally recognized as the best diagnostic system available today, it cannot be used for a screening or periodic monitoring of potentially malignant oral disorders, precancerous lesions or suspicious lesions that could evolve, because it is invasive and could leave a scar that could alter the outcome of future diagnoses. A screening test is not intended to be diagnostic but aims to capture patients with such abnormal oral findings and to accelerate the referral and application of more specific diagnostic procedures by a specialist.

Human saliva has been considered a valuable source for protein or nucleic acid biomarkers for various infections, systemic and non-systemic diseases (63). Exfoliated cancer cells may release proteins and free molecules representing gene expression changes associated with tumor development into the saliva, thus salivary proteins provide a strong option for development of non-invasive, point-of-care assays for screening/early detection of oral cancers. Among the proteins verified, CD44, S100A7 and S100P showed significant potential for use as early detection markers in patients with dysplastic leukoplakia and OSCC (64).

In a previous study, Lichieh Julie Chu et al. identified matrix metalloproteinase-1 (MMP-1) as one of the most promising salivary biomarkers for OSCC and developed a sensitive ELISA for MMP-1 with good performance in detection of OSCC. More recently, they developed a time-saving rapid strip test (RST) and demonstrated that salivary MMP-1 levels measured using RST and ELISA were highly comparable and both assays could effectively distinguish between OSCC and non-cancerous groups (65).

The technique proposed in the present paper aims to be non-invasive and capable in the future of phenotyping suspicious lesions based on the number and quantitative level of tumor markers present on a lesion. Moreover, the source in which to search for the markers has been carefully evaluated, preferring cytobrush samples containing fragments of tissue and cells directly from the suspicious lesions where the markers would be much more concentrated than samples of blood, saliva, or other organic fluids. Such a technique aims in the future to become valuable as an aid for obtaining an early diagnosis and help clinicians in setting up a prompt therapy in case of malignant positivity. This technique was also designed to be also rapid, cost effective and patient-side. The small number of cases does not allow the authors to indicate the materials and methods adopted for the present study as a new diagnostic tool that can be used for point of care screening, although, from a statistical perspective, the number of samples is already significant enough to validate our observations. Therefore, the extremely positive and encouraging results of our study require the future evaluation of larger samples to confirm its potential value for the early detection of oral cancer and the assessment of disease progression.

## 5 Conclusions

In conclusion, our study strongly suggests that the search of selected tumor markers on brush biopsy could be successful for the detection of oral cancers and precancers.The proposed technique consists in a rapid non-invasive possible alternative or companion examination to the gold standard (biopsy). This procedure quantitatively and qualitatively evaluates the presence of six markers proven to be associated with neoplastic transformation. The results showed how each of the six markers selected is significatively overrepresented in the neoplastic lesion compared to the healthy tissue, therefore the combined evaluation of the six markers could be able to determine the presence of neoplastic formations with high accuracy.

Statistical power analysis showed that the study sample has the necessary size, anyway it would be very interesting, in future works, to further expand the sample population to confirm the effectiveness of the proposed test on a large statistical base. If the results of the present study are confirmed for a large sample as well, this technique could be applied to define the phenotype of precancerous lesions based on the markers expressed, paving the way to an innovative method of screening to prevent oral cancer.

## Data availability statement

The original contributions presented in the study are included in the article. Further inquiries can be directed to the corresponding authors.

## Ethics statement

The studies involving human participants were reviewed and approved by ethics committee of the Liguria Region, Genova, Italy (n. 326/2018 and n127/2022-DB id12223). The patients/participants provided their written informed consent to participate in this study.

## Author contributions

FR, ADR, MG designed, performed research, and interpreted data; RoP, ReP, FG, PI, FL, BB provided patient’s profile; GT, MG, SP performed statistical analyses, FB, FDG revised the article; AR, EM designed and performed research, interpreted data, wrote the article. All authors contributed to the article and approved the submitted version.
